# A flexible user-interface for audiovisual presentation and interactive control in neurobehavioral experiments

**DOI:** 10.12688/f1000research.2-20.v2

**Published:** 2013-06-06

**Authors:** Christopher T Noto, Suleman Mahzar, James Gnadt, Jagmeet S Kanwal

**Affiliations:** 1 Department of Neurology, Georgetown University, Washington DC, 20057, USA; 2 Department of Physiology and Biophysics, Georgetown University, Washington DC, 20057, USA; 3 Current address: Faculty of Computer Science and Engineering, GIK Institute, Topi, 23640, Pakistan; 4 Current address: NINDS/NIH, Systems and Cognitive Neuroscience, Neuroscience Center, Bethesda MD, 20892, USA

## Abstract

A major problem facing behavioral neuroscientists is a lack of unified, vendor-distributed data acquisition systems that allow stimulus presentation and behavioral monitoring while recording neural activity. Numerous systems perform one of these tasks well independently, but to our knowledge, a useful package with a straightforward user interface does not exist. Here we describe the development of a flexible, script-based user interface that enables customization for real-time stimulus presentation, behavioral monitoring and data acquisition. The experimental design can also incorporate neural microstimulation paradigms. We used this interface to deliver multimodal, auditory and visual (images or video) stimuli to a nonhuman primate and acquire single-unit data. Our design is cost-effective and works well with commercially available hardware and software. Our design incorporates a script, providing high-level control of data acquisition via a sequencer running on a digital signal processor to enable behaviorally triggered control of the presentation of visual and auditory stimuli. Our experiments were conducted in combination with eye-tracking hardware. The script, however, is designed to be broadly useful to neuroscientists who may want to deliver stimuli of different modalities using any animal model.

## Introduction

In neurophysiological research, correlating neural signals driven by stimulus presentation and behavioral response needs to be completed within a limited time frame, generally less than 2 hours when conducted with non-human primates. This requires effective and efficient control of presentation of stimuli, acquisition of data, and monitoring of behavior for reward and task progression. Behavioral neuroscientists have to continuously struggle to both keep up with technological advances to accelerate data throughput and to customize stimulus delivery and data acquisition systems to do cutting-edge research. This adds to the burden of labor-intensive electrophysiological recordings from single or multiple neurons in awake-behaving animals, which nevertheless continues to be one of the most reliable and useful ways to understand neural computations and function. Stimulus presentation paradigms may also need to be routinely modified to conform to the goals of an experiment. All of this has to be accomplished with the constraint of maintaining the experimental animal in a healthy condition until the experiment has run its course, which may take from weeks to months. Moreover, user requirements, dictated by the scientific data and state of knowledge, are a moving target that makes it difficult for for-profit vendors to meet all the needs of their customers. Laboratory heads are frequently faced with the task of either hiring a permanent programmer at the cost of tens of thousands of dollars in annual salary to create and maintain a new program, or abandoning a particular line of experiments that scientifically may be the right direction in which to proceed. Even the choice of hardware and software packages that laboratory personnel could interface with and manipulate easily largely depends upon the available expertise of those working in the laboratory and frequently shifts with the departure of key personnel.

**Figure 1.**
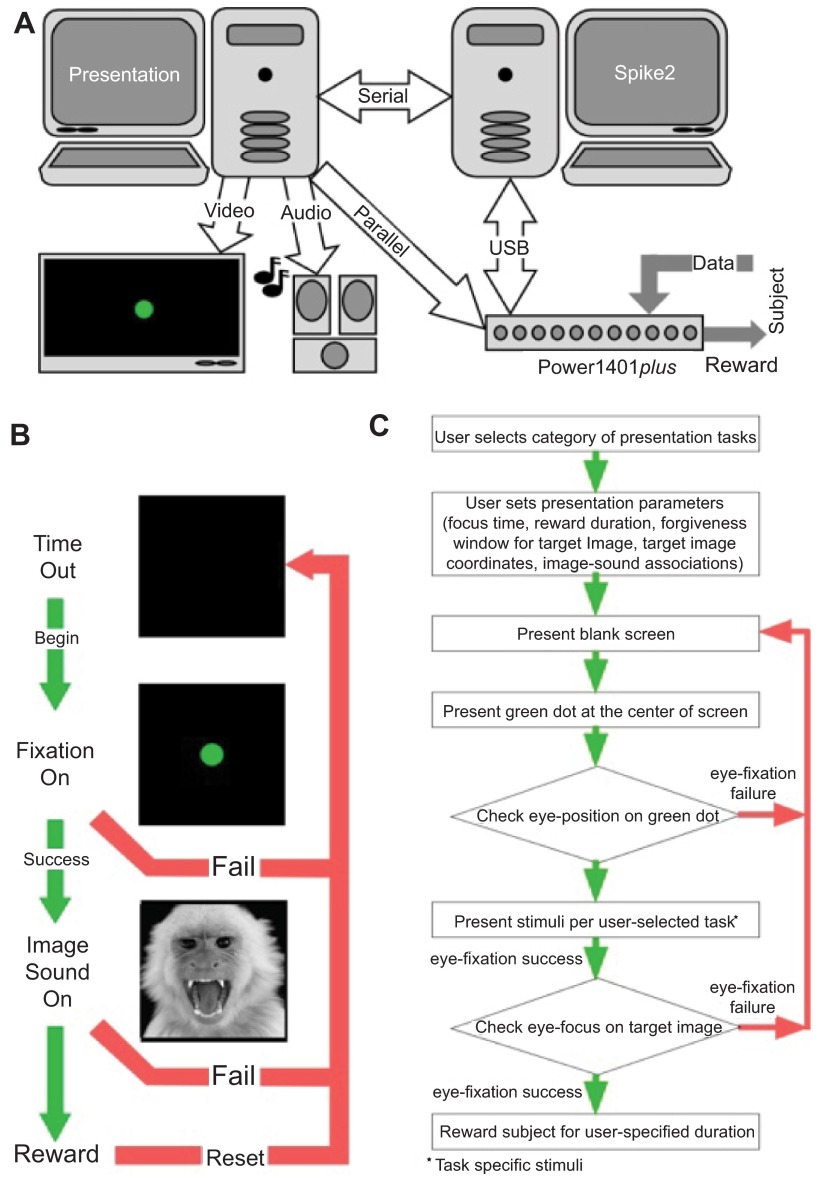
System hardware connectivity and experiment flow chart. **A**. The system is divided into three levels: Data Acquisition and Behavioral Monitoring, Stimulus Presentation Control, and Stimulus Presentation. **B**. Typical flow of experiment based on performance of subjects. **C**. Logical flow chart for behavioral tasks used in the training and testing paradigms.

To effectively meet our own needs for the study of gaze control in response to the presentation of audiovisual stimuli, we developed a user-interface that provides a template for others facing a similar challenge. Specifically, we describe an experimental design that uses a custom-written script for controlling communication between Presentation software (Neurobehavioral Systems, Inc., Albany CA) package and data acquisition hardware (Cambridge Electronic Design, Ltd., Cambridge, UK) along with vendor-provided Spike2 software. Each package runs independently on separate personal computers ( Figure 1A).

Rapid eye-movements, or saccades, channel important visual information into the association and prefrontal cortex where it is integrated with previous knowledge to take decisive action ^1– 3^. Much research effort has gone into validating the role of the superior colliculus (SC) ^4– 7^ and cortical areas, such as the frontal eye fields ^2^ and lateral intraparietal areas ^1^, in the control of eye movements during visual saccades. Less is known about the kinematic properties and control of auditory priming of saccades ^8, 9^ and virtually nothing is known about the modulation of visual saccades by contextual auditory information. Clearly, sensory recall of auditory objects as well as error-correction and decision mechanisms underlying memory-guided saccade initiation or head orientation need to be invoked ^9– 11^. It is less clear, however, where in the brain these two functionally distinct mechanisms might converge ^12, 13^.

We used our newly developed audiovisual presentation and control scripts to acquire new data on responses to auditory and visual stimuli in a reward-driven behavioral task that involved tracking eye movements in a nonhuman primate. Our goal was to facilitate the exploration of neurons that integrate multimodal sensory information from naturalistic stimuli to elicit adaptive behavior. As a first step, we trained monkeys to associate relatively novel sounds, including animal vocalizations, with images that were also considered novel for monkeys maintained in a captive environment. To begin to explore the pontine circuitry creating such associations as well as eye movements, we first used species-specific calls to identify complex auditory stimulus-driven neurons in the IC, and naturalistic images to identify visual and saccade-driven neurons in the SC. This narrowed our search space for finding audiovisual neurons, located potentially at the boundary region between the IC and the SC, and testing if reward modulated their activity. We focused on eye movements as the adaptive behavior since these can be accurately tracked, provide a rapid response, and are controlled by neural activity within the SC ^4– 7^.

## Materials and methods

### Materials

We used two software packages, Presentation (Neurobehavioral Systems, Inc., Albany CA) and Spike2 (Cambridge Electronic Design, Ltd.) in conjunction with data acquisition hardware (Power1401 *plus*, Cambridge Electronic Design, Ltd.), to control stimulus presentation based on our subject’s behavior. This required communication between Presentation and Spike2 software via serial and parallel ports to either advance or terminate a subject’s task in real-time based on either correct or incorrect behavioral responses, respectively. Data acquisition at a relatively high sampling rate (0.1 ms resolution) by the Power1401 was performed concurrently with stimulus presentation and behavioral monitoring. Our design integrates hardware that is either routinely available in a neurophysiology laboratory or commonly available from vendors ( Table 1 and Figure 1A). Presentation software is readily available from Neurobehavioral Systems for on-line download ( http://www.neurobs.com). We chose Presentation because of its large, comprehensive scripting language and intuitive user interface, and because the software allowed a simple method to communicate via both serial and parallel ports of a personal computer running a Windows operating system. The software itself is easy to use and numerous example scripts make the language easy to learn. Spike2 and the Power1401 *plus* are available for purchase from Cambridge Electronic Design ( http://www.ced.co.uk/indexu.shtml). We chose Spike2 and the Power1401 *plus* because of its extensive scripting capabilities, ease of use, and inherent control of data acquisition (here, an analog-to-digital converter cycled through the incoming signals at 1 MHz), independent sequencer control, and straightforward manipulation of both parallel and serial ports (see http://www.ced.co.uk/pru.shtml for hardware specifications). Each software package runs independently on its own personal computer to avoid compromising processor resources. Under Presentation control, video is output by a Radeon 9250 video card on a 55” Visio flat panel HD TV and sound is output using a SoundBlaster audio card by a Bose speaker system. A 16 ms error is inherent in the presentation of visual stimuli due to the 60 Hz refresh rate of the LCD monitor. For experiments designed to perturb subconscious elements of the visual system, display delays could be accounted for by additional code written into the scripts discussed below, or an LCD display may be substituted with some other form of imaging, e.g. a fast stepping motor turning a vertically oriented circular slide tray.

**Table 1.** A list of computer and electronic items used in our stimulus presentation, behavioral monitoring, and protocol control system (standard data acquisition and electronic equipment used for electrophysiology is not listed here).

Hardware components	Software components	Approximate cost
Dell Dimension Computers	x	$800
Vizio, E55VL 120Hz LCD HDTV	x	$500
Bose, Companion 3 series II Speakers	x	$550
x	Presentation – Neurobehavioral Systems, Inc.	$250/year
Power1401 *plus* – Cambridge Electronic Design, Ltd.	Spike2 – Cambridge Electronic Design, Ltd.	$9,500

### Animal care and preparation

Three Rhesus monkeys ( *Macacca mullata*; 2 males and 1 female) acquired from a research facility at Wake Forest University, were available during various stages of testing and data acquisition for the development of protocols described here. Compatible animals were housed in paired grooming/contact cages (~2.5 cubic meters), in a room with a light and dark cycle set by an automatic day/night timer (light from 6AM to 6PM daily) and with full view of colony mates in a large open room. Cages were continuously equipped with swings, mirrors, foraging devices and/or small toys. Daily care and medical maintenance of the animals, including a balanced diet of dry food formula, vegetables and fruit, were routinely provided. Environmental enrichment for the monkeys included playing of natural sounds, radio or TV and daily handling, mock grooming and socialization by laboratory personnel.

### Surgical procedures: eye coil implantation and neural recordings

Animals were prepared for participation in experiment by performing two surgeries. For the first surgery, we implanted a head restraining device and one scleral eye coil. With the head secured in a stereotaxic device, a 5 cm midline incision was made in the scalp. Periosteum and muscle was retracted using blunt techniques and the calvarium scraped free of soft tissue. A 3 cm stainless steel bar, which fits a head restraining apparatus of the primate chair, was attached vertically to the calvarium using surgical stainless steel screws and a stainless steel recording chamber anchored to the skull using screws and a mound of acrylic bone cement ^14, 15^. The screws are mounted into small burr holes in the bone and buried in the bone acrylic along with the head post and electrical connectors. A scleral eye coil was implanted on one eye. Briefly, the conjunctiva was cut near the limbus and reflected to expose the sclera. A coil made of three turns of Teflon-insulated wire was sutured to the sclera using 6-0 Vicryl, and the conjunctiva was sutured back over the coil. The ends of the coil wire were led out of the orbit subdermally to the acrylic cap where they were attached to a small electrical connector. One week post-surgery, we began a daily task-specific training regimen. Once training proceeded to an acceptable level, generally within a few months, another aseptic surgery was performed to implant an eye coil on the second eye and one or two stainless steel recording chamber(s) were mounted into the head cap under stereotaxic guidance. The acrylic overlaying the appropriate portion of the skull was removed using dental burrs in a hand drill and a 15 mm craniotomy was made. Stainless steel recording cylinders were placed over the craniotomy and cemented into place with bone or dental acrylic. The sterile interior of each cylinder was secured with a threaded Teflon cap having a pressure-release vent.

Post-surgical maintenance included prophylactic antibiotics for 7 days (Baytril, daily 2–5 mg/kg) and 2–5 days of narcotic analgesics (buprenorphine, 0.05–0.1 mg/kg BID) followed by 3–5 days of acetaminophen (5–10 mg/kg). Flunixin, a non-steroidal anti-inflamatory agent, was administered for 1 to 3 days (0.5–1 mg/kg). We also monitored body weight and food/water intake daily, and performed maintenance of the skin margin and cleaned the recording cylinders.

### Behavioral training

During the behavioral training, the monkeys sat in the Plexiglass primate chair within a cube of magnetic field coils. To avoid recording of eye movements being confounded with head motion and to stabilize the head while electrodes are inserted in the brain, the head was restrained painlessly by clamping the head post to a device on the chair. To motivate the subjects to perform adequately, for five days per week they received their daily fluids as reward for proper behavior. When daily training or experiments are terminated prematurely, fluids are supplemented up to the normal daily level for that subject. Fluid intake was monitored and recorded daily. Additionally, pulpy fruit or vegetables were used to reward good behavior when returning the animal to the home cage.

Using standard behavioral shaping procedures, the animals were trained to fixate and to follow small visual or auditory stimuli by rewarding them with a drop of fruit juice from a gravity-fed “straw” for successfully completing each series of eye movements defined by the presentation of the stimuli. Training and experimental procedures were performed for no longer than 5 hours per day, usually for 1–3 hours. Animals exhibiting discomfort were readjusted within the chair or returned to their home cage. The daily manipulations for the animals did not produce pain or distress. The cooperative demeanor of the monkeys gives us reason to believe that they find the laboratory situation stimulating and the social interaction with the investigators satisfying.

All surgical and experimental procedures were performed in accordance with federal and institutional guidelines on the care and use of laboratory animals as part of protocols approved by the Georgetown animal care and use committee (protocol # 09-025).

### Stimulus display and trial design

Figure 1B shows what is displayed on the screen and the actions of the subject in response to the presentation of a visual stimulus. Figure 1C is a logical flow diagram to show the various steps listed as 4 tasks in the experimental scheme. The tasks are described as follows:

1. Association task:

A sound is played and the associated target image is simultaneously presented at the center of the screen. In our experiments, short (1 s) tone bursts and natural sounds (communication calls) were presented at stimulus levels of ~80 dB SPL (decibels of sound pressure level).

2. Left-right-association task:

A sound is played and an associated target image is simultaneously presented centered at a horizontal location a user-selected distance from the center of the screen, either on the left or on the right side (the decision to present left or right is decided randomly at run-time).

3. Single distracter task:

(a). A sound is played and at the same time a “green dot” is presented at the center with simultaneous presentation of the associated target image and a distracter image on either sides of the circle. The position of images is decided randomly at run-time.

(b). The target image and distracter image are retained on screen and eye-focus is monitored.

4. Multiple distracters task:

(a). A sound is played and at the same time, a “green dot” is presented at the center with simultaneous presentation of an associated “target image” and multiple (user selected) distracter images at user-specified locations on the screen. The position of the images is deliberately kept fixed in this task.

(b). The target image and distracter image are retained on the screen and eye-position is monitored.

### Experimental design

**Figure 2.**
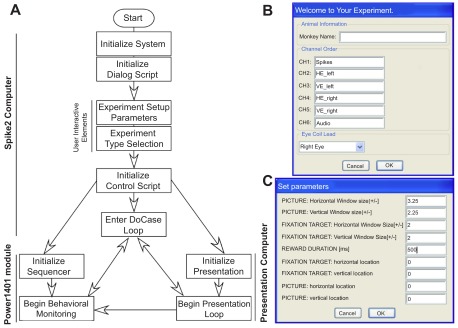
Details of script and user input. **A**. Flow chart showing the design of experiment control sequence shared between the four scripts. **B**. The initial interactive user-interface used to collect basic information about the experiment set-up. **C**. User-interface used to collect the initial parameters for behavioral monitoring of subjects during experiments.

Running the script described in Figure 2A provides a user-interface in Spike2 that begins a cascade of dialog boxes that request information relevant to the experiment (e.g. subject name) and the basic parameters needed to monitor the behavior of the subject (e.g. detection window size, reward duration). After supplying the basic information ( Figure 2B), a list of experimental scenarios is presented to the user in order to select the condition a subject will face. We have programmed a number of saccade-related tasks that use one (or more) of eight audio stimuli to direct our subject’s behavior to learned associations of visual images. A check box arrangement indicates a combination of stimuli the user intends to use in the experiment. As well, a number of timing variables (‘Time to get on Target’, ‘Initial Fixation Time’, ‘Fixation Time for Reward’) are adjustable by the user. Clicking the ‘OK’ button collapses the association-training dialog box allowing the user to hit the ‘Run’ button to initiate the scenario or to select a different scenario. From this point forward, the parameters dialog, the experimental scenario dialog, and a quit option are always available as buttons to the user on the Spike2 program interface. Selecting another experimental scenario automatically names and saves the current data file while initiating data collection into a new file for the newly selected scenario. Clicking on the ‘quit’ button saves the current data file, terminates the presentation of the ongoing stimulus to the subject and ends execution of the Spike2 script.

### The association-training paradigm

**Figure 3.**
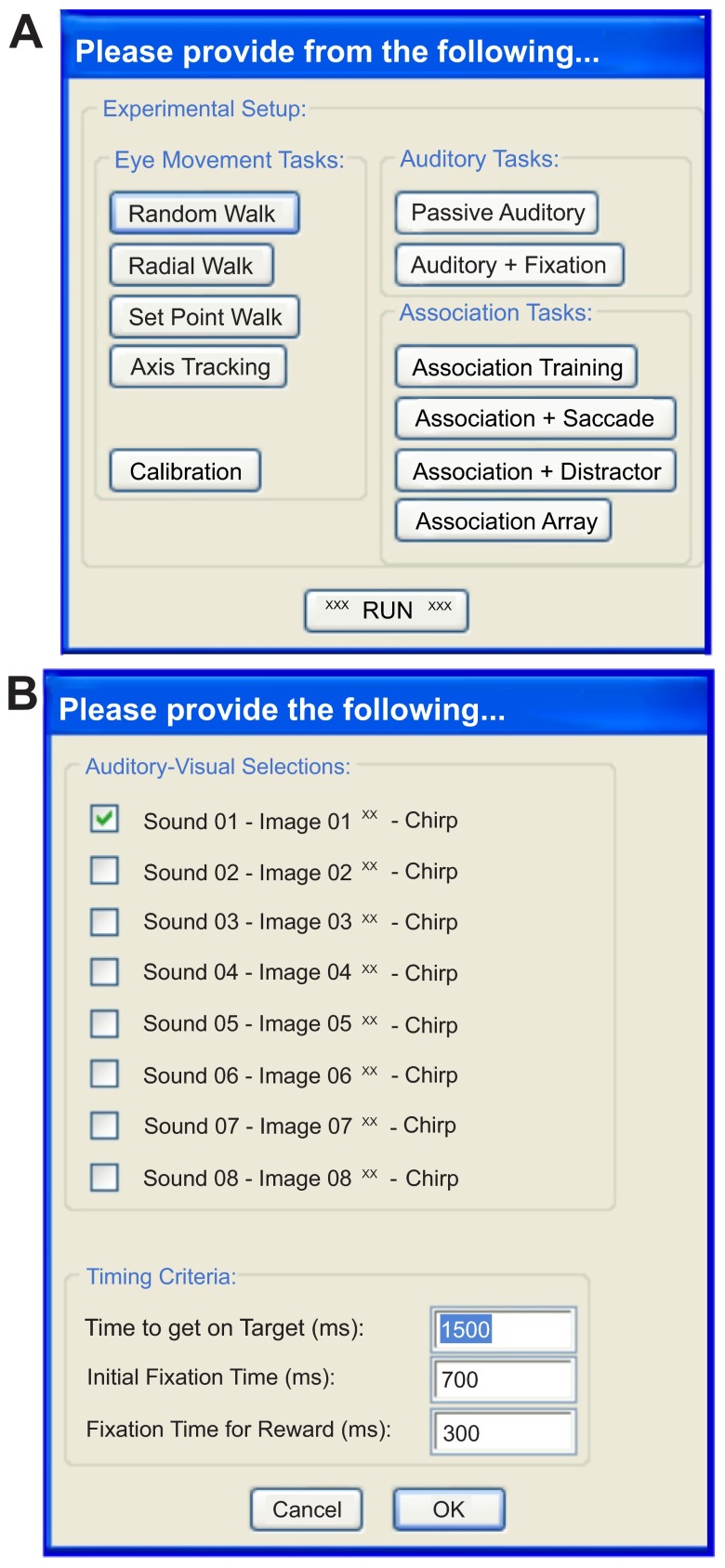
Menu-driven user interface. **A**. The list of proposed paradigms users may select from to start a session; currently available are Calibration and the Association Tasks, and include “walk” tasks that were not implemented in the present version. “Walks” are saccade tasks designed to use a single target that appears on a black screen in various locations, moving in patterns ascribed by the selection buttons and subsequent dialogs boxes that may be added by the user. They can be used to train the animal and record metrics of their eye movements. **B**. User-interface that allows for selection of the auditory-visual pairing used during the association training paradigm and the timing criteria necessary for successful completion of the task.

Although quite simple, our ‘Association Training Paradigm’ allowed us to illustrate the inner workings of 1) the “Spike2 control” script, 2) the “sequencer script”, and 3) the “presentation script” as they operate across all the current scenarios available to the user. Before going on, we should discuss what we expect from the program and subject, so we can better discuss the interweaving functions of these three scripts. Figure 1B shows the progression of stimuli if the subject succeeds across all phases of the trial or fails at any time in the trial. This task has three phases: 1) an initial black screen or timeout screen, 2) an initial fixation target, and 3) test stimulus presentation. During phase 1, behavior is not actively monitored. The duration of the timeout is set to 2 seconds in the presentation script. At the inception of phase 2, the sequencer acting through the script loaded to the Power1401 memory begins monitoring eye position. The subject must first acquire the target and maintain gaze on the target within a small “forgiveness” window for the user-defined epoch of time. Successful fixation of the target advances the scenario to phase 3 by a command issued first from the sequencer to the Spike2 ‘control’ script and then from the ‘control’ script to the presentation script. Failure results in a reset to the black screen and a brief timeout using the same flow from sequencer to presentation script. A response token is sent directly back from the presentation script to both the ‘control’ script and sequencer ensuring that all three scripts remain synchronized. Phase 3 consists of the presentation of our test stimuli, here the co-presentation of an image and sound. Successful fixation of the image within a forgiveness window, equal to the size of the image and for the user-defined time, initiated by a dialog box shown in Figure 3A, results in the delivery of a reward to the subject as commanded by the sequencer. Successful fixation or failure to look at the image commands a reset of the experimental process to the black screen for a 2 second refresh period.

### Script components

Our stimulus delivery and data acquisition package consisted of four primary components that operate in conjunction with one another. A “sequencer” script written from within Spike2 is loaded to the Power1401 for real-time monitoring of eye position and saccades. Sequencer scripts (included in the “Sequencer Files” file below) are ultimately responsible for issuing commands that direct progression through a task and to reward the subject. Two scripts (included in the “Spike2 Control Scripts” file below) operate in the Spike2 software environment. The first script controls the interfaces into which a user inputs relevant control parameters. The second script provides the functional control between the scripts running on the Spike2 computer and script running on the Presentation computer. One script (included in the “Presentation Scripts” file below) runs in the Presentation software environment commanding the output of stimuli and communicating by connections of the parallel line (to the Power1401) and the serial line (to the computer running Spike2) a time-stamp indicating when the presentation script commands the presentation of a stimulus.

### Sequencer script

The sequencer script downloaded to the Power1401 module runs using an independent clock ticking at 1 µs from the Spike2 computer, but communicates with it through a high-speed USB port. The sequencer script consists of two parts: 1) the initialization section and 2) the monitoring section. The initialization sections load the user-defined variables set while interacting with the dialog boxes created by the ‘interface’ script. In our example of the association-training scenario, the variables loaded are the edges of the forgiveness window, the three timing criteria (time to get on target, initial fixation time, and fixation time for reward), and reward pulse duration. The sequencer cannot act on these values directly so we convert them to sequencer-relevant values. The edges of the forgiveness window are converted from the user-defined values in degrees to digital-to-analog converter (DAC) values. The timing criteria and reward pulse duration are converted from milliseconds to sequencer steps per ms. The monitoring section is made up of the same number of sections as the scenarios or situations (here three), each with specific tasks. The first task checks that the subject acquires the fixation target after it is presented within the user-defined epoch of time. The state of fixation, success or failure, is sent to the Spike2 ‘control’ script. If the sequencer determines the subject has worked within the task bounds, the sequencer steps to the next phase of the monitoring section and waits for a confirmation that the scenario has advanced from the ‘control’ script. The second task checks that fixation is maintained on the target for the specified time. Once again, information about the state of fixation, either success or failure, is sent to the Spike2 ‘control’ script. If the sequencer determines the subject has worked within the task bounds, the sequencer steps to the final phase of the monitoring section and waits for a confirmation that the status has advanced from the ‘control’ script. The final phase operates exactly as the second phase except that if the subject complies, a reward pulse is sent from the Power1401 to a reward delivery system though one of the digital I/O ports. Regardless of whether the trial is deemed a success or failure, the sequencer returns to the initialization section and resets the variables to their initial user-defined state. The process loops with each trial.

Spike2 sequencer script files. Multiple sequencer files necessary for monitoring and rewarding correct animal behavior during the various tasks

### The Spike2 ‘control’ script

The ‘control’ script runs in the background on the Spike2 computer and uses the first bit of the COM-1 port input to communicate with the Presentation computer and controls advancement through the Presentation script. The serial line conveys the hexadecimal representation of the words (descriptors and terminators) used to call images and sounds and response tokens between acquisition and presentation computers, respectively. This bit was opened, written to, and closed by the respective portions of the scripts running on the acquisition or presentation computers. Each task consists of a looping “do case” function with progress through the function determined by the fixation state passed from the sequencer. In our example of the association-training task, there exist three fixation failure situations and three successful fixation situations. The first failure scenario occurs when the screen is black and the subject has no target to fixate. The script simply calls for the presentation of the fixation target by issuing a command to the computer running Presentation. The second and third failure situations are similar and initiate a command for the presentation of the black screen to the subject. The first success case assumes that the subject’s gaze is directed toward the fixation target’s location when there is none present. In this situation, the script calls for the fixation target to be presented, just as in the first failure case. The second success situation calls for the Presentation script to display the test stimulus. The third success scenario initiates a reset of the screen to a blank (black) display by the Presentation script. Following each call to the Presentation computer, the ‘control’ script listens for a reply on bit 1 of the COM-1 port. Upon receipt of the response the “do case” state is returned to the sequencer to allow advancement through the monitoring sections of the script ensuring proper stepwise alignment of all three scripts throughout the task.

Spike2 control script files. The two control scripts used to interact between users and the presentation script as well as the configuration used to collect data.

### The presentation script

The Presentation script running on the Presentation computer acts as a slave to the Spike2 ‘control’ script receiving instructions and replying through the first bit of the COM port. This script has three primary sections: 1) the video monitor setup, 2) image and sound object creation, and 3) the experimental loop. The first section of this script requires the user to predefine the current display properties (resolution and color depth) including the height and width of the monitor, and distance of the subject to the screen. In this way, Presentation calibrates itself so that target and image positions may be stated in degrees and drawn at the appropriate size. The second section predefines all the potential objects that may be called during the experiment after selection via a dialog box ( Figure 3B) and their association, if any. For example, our fixation target is a small green dot. We have created an object (e.g. named ‘greendot’) that holds all the relevant information about how presentation draws our fixation target (e.g. dot size, color of the background, etc.) when a call is made to the object. In the third section, the Experimental Loop monitors the COM-1 port for communication from the Spike2 computer. This loop is largely comprised of “if, then, else” statements. Each communication from the ‘control’ script is pre-defined so that when the ‘control’ script shunts words and terminators (e.g. ‘grendot\n’) to the Presentation computer, the Experimental Loop recognizes the word (grendot) and terminator (\n) and falls into the appropriate “if” statement. In the case of the ‘grendot\n’ combination, the “if” statement calls for our object ‘greendot’ so that the fixation target is displayed on the monitor, and at the same time triggers a reply to the ‘control’ script on the COM-1 port and to the Power1401 on bit 8 of the parallel port (a 1 ms low-high-low transistor-transistor logic (TTL). The script then returns to the loop, listening for the next command from the Spike2 computer. In this way, each object may be called in any sequence as commanded by the Spike2 ‘control’ sequence. In the case of our example, the next word that the loop would receive would be ‘SndPICn\n’. Similarly, the loop falls into the appropriate “if” statement, displays the test stimulus, replies to the Spike2 computer and Power1401, and returns to the loop.

Presentation script files. The single necessary presentation script needed to display images and sounds upon command from the Spike2 control script.

Associated sound and target image files. Paired sound and image files necessary for running all association tasks, frequency tone ranges used for testing frequency tuning of neurons (not discussed in the text) and species-specific communication calls.

### Data acquisition and analysis

Up to five days a week, a two-hour neural recording period occurred between 10AM and 4PM to ensure overlap with veterinary staff hours. Animals were moved from their home cage to an adjacent room for neural recording sessions while seated comfortably in a primate chair. In the recording room, the animal’s head is fixed facing forward, in full view of the LED (light emitting diode) monitor set 48 inches in front of them with the center of the screen at the approximate height of the animals straight ahead gaze. Extracellular neuronal recordings were made using standard electrophysiological methods in behaving subjects using fine wire tungsten microelectrodes (31 gage, Microprobe, Inc.) mounted in a guide tube of stainless steel hypodermic tubing ^16^. Transdural penetrations were made by a hydraulic microdrive (FHC, Inc.) advancing a tungsten electrode through the bore of a 21 gage hypodermic needle mounted in a micropositioner that attaches to the outside of the chronic recording cylinders on the animal’s head. Neuronal activity was recorded on the hard drive of the data acquisition computer running the Spike2 control scripts via a high impedance amplifier system (AMC Systems, Inc.). We collected one channel of raw neural signal at either 25kHz or 50kHz, four channels corresponding to horizontal and vertical eye position at 1kHz, one auditory channel at 25kHz, and one channel of timestamps at 10kHz, generated on-the-fly during acquisition of data using an adjustable threshold set on the channel collecting the neural signal, for spike times. Digitizing the raw neural signal allowed for post-hoc analysis using the Spike2 software that provides software window discriminators and level detectors as well as various forms of waveform analysis including template matching and spike sorting, using PCA algorithms.

Data analysis and recording was conducted using Spike2 software (Cambridge Electronic Design, Ltd.). Custom-written scripts were used to build raster plots and peristimulus – time histograms (PSTHs) for display of processed data for well-isolated single units whenever possible. Only sample data from single or few-unit activity are provided here to demonstrate feasibility for the purposes of this project, which was designed for development of experimental control procedures.

## Results and discussion

**Figure 4.**
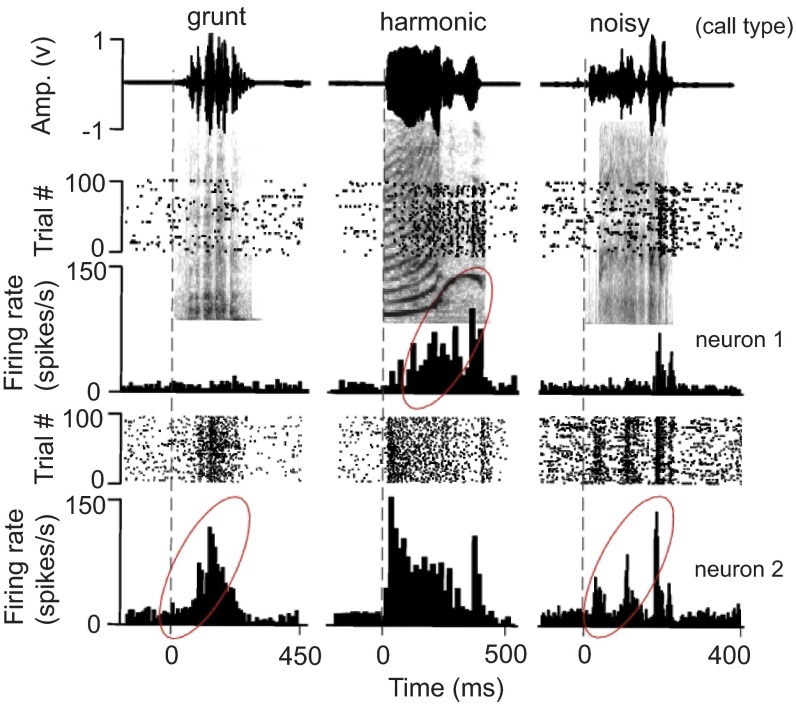
Responses of collicular neurons to communication sounds. Amplitude envelops (top) and raster and PSTH (10 ms bins) plots superimposed on call spectrographs of three different call types (grunt, harmonic, noisy) to show the response of 2 neurons (neuron 1 is from a female and neuron 2 is from a male) in the monkey inferior colliculus. Each call presentation was repeated 40 times per histogram. Grey vertical dotted lines indicate sound onset. Note the response build-up to the third predominant amplitude modulation in the last call. Average first peak response latency to calls was 20.9 +/- 3.5 ms (n = 10). Responses with a potential for temporal facilitation are enclosed by ellipses, although response enhancement may also depend on the basic acoustic patterns within complex sounds or on amplitude tuning. Calls were downloaded from the following web site: http://www.soundboard.com/sb/Rhesus_Monkey_sounds.aspx.

As proof of concept, we present here a number of behavioral and neural responses from various brain structures that are activated in response to naturalistic stimuli presented within our experimental set up. To reiterate, we were primarily concerned with capturing 4 basic types of neural responses: 1) visual, 2) auditory, 3) saccade, and 4) reward-driven. This analysis utilizes the timestamps placed in the data files by the presentation script’s 1 ms TTL pulse sent to the Power1401 during data acquisition. Neurons were recorded from the midbrain in the putative inferior and superior colliculi (IC and SC, respectively) of one of our nonhuman primate subjects. Figure 4 describes typical neural responses in the IC following the presentation of complex communication sounds or “calls” that contained acoustic features preferred by the neuron ^17– 20^. Of the ten neurons from which electrophysiological activity was recorded, all responded to at least one of the seven sounds presented. As an example, Figure 4 shows the response of two neurons from the same animal to the same three sounds. We found that each sound produced a distinct temporal response pattern. These patterns could range from no or transient increases in the overall firing rate (upper left panel) to intense phase-locked responses to acoustic features within a call (lower right panel).

**Figure 5.**
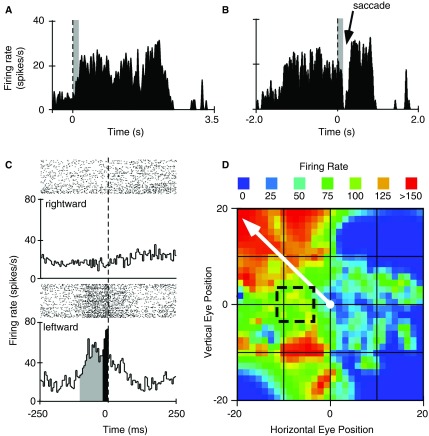
Fixation and saccade-related neural activity in the intermediate superior colliculus. **A**. Summed histogram from multiple trials triggering visual stimulus-induced activity in a “fixation” neuron located at the rostral pole and superficial layers of the superior colliculus (SC). Electrophysiological responses (timestamps for spikes) were aligned to the time at which the subject acquired the fixation target to begin the trial. **B**. Saccade-triggered transient suppression of neural activity in a different neuron located within intermediate layers and caudal to the fixation-neuron in the same animal. Dashed vertical line is at time zero for stimulus presentation in “A” and for target fixation in “B”. The width of the grey bar indicates the neuron’s visual delay (>20 ms). **C**. Raster plots (above) and binned profile of summed response (below) to compare neural activity during rightward (top panel) and leftward (bottom panel) saccades. Grey bins indicate the build-up phase while the black bins indicate the burst phase of the neuron. Dashed vertical line indicates saccade onset. **D**. Heat map of saccade-related neural activation. Black box enclosed by dashed lines indicates position of the target image relative to central gaze. Solid white arrow represents the vector for the preferred saccade as indicated by firing rate of the neuron.

The neuron shown in Figure 5A and 5B illustrates the characteristic visual activity one expects to find while recording from rostral-superficial layers of the SC ^21^. Once gaze was directed to position the eyes within the receptive field of this neuron we observed steady, low-rate firing within ~20 ms. In this example, the subject was required to make a saccade to capture a sound-associated image. After the fixation target was extinguished and the target image was presented in the peripheral field of vision, the neural response declined and resumed only when the eyes were positioned again on the target image. The neuron shown in Figure 5C and 5D fits the characteristics attributed to neurons of the intermediate layers of the SC ^22, 23^. Namely, a 60–80 ms build up in activity followed by a burst of spikes just prior to the initiation of direction-dependent saccades to our visual stimulus. Examination of the neural data collected during “spontaneous” eye movement behavior shows that this neuron preferred saccade vector (>20 degrees amplitude, 137 degrees angle), which is well off the axis of our stimulus.

Example neural recording files Example data collected in the IC and SC showing auditory, visual and saccade-related activity, as well as reward-related activity.

Of the total population of neurons studied in the SC by Jay and Sparks ^12^, 79% showed saccade-related bursts prior to eye movements to either visual or auditory evoked target stimuli suggesting that saccades evoked by either stimulus share a common efferent pathway to generate the movement. Meredith *et al.* ^13^ recorded 113 neurons in the SC (82/113 were auditory-visual neurons) of anesthetized cats during presentation of single and temporally overlapping sensory stimuli. Peak response in neural firing to multisensory signals occurred when stimuli were presented concurrently, with the second stimulus starting <100 ms from the first. Since then, research has shown that in the deep layers of the SC, most neurons respond to both visual and auditory stimuli; 99 of 121 SC neurons showed significant alteration in firing rates due to eye position ^12^.

**Figure 6.**
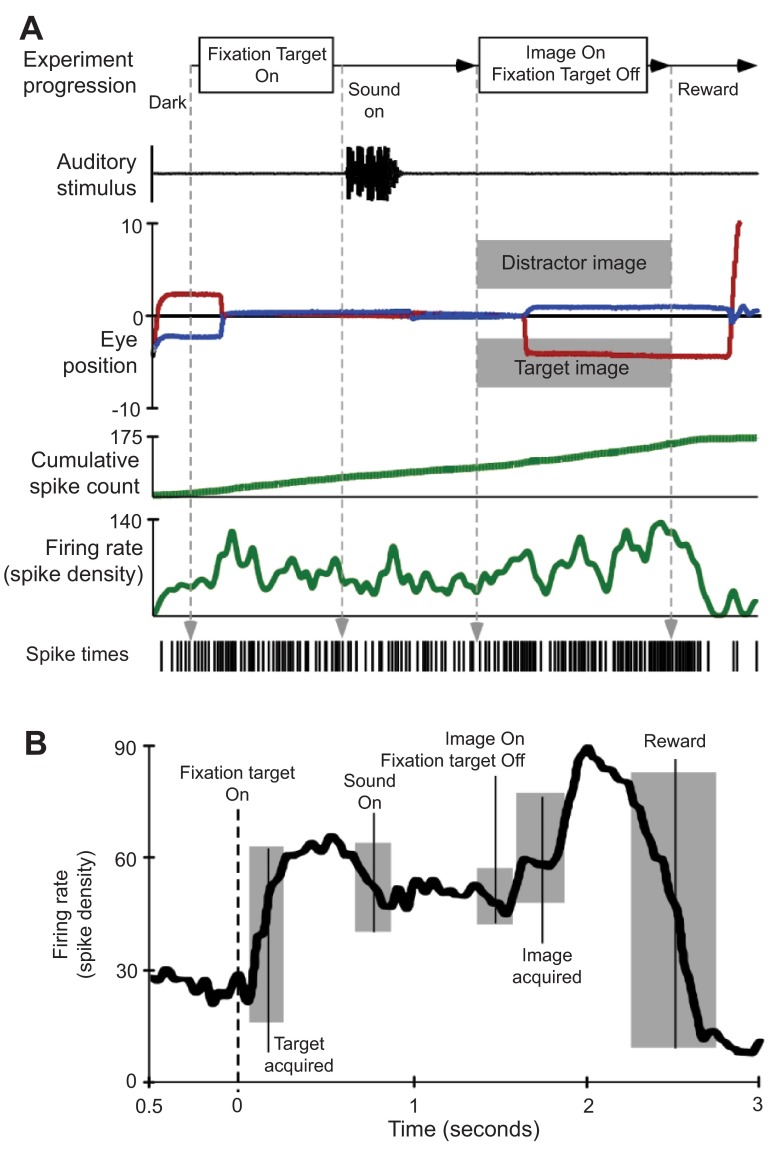
Reward induced neural activity. **A**. Single experimental trial illustrating stimulus presentation and related neural activity. During these trials, the subject was rewarded for successful discrimination of a sound-associated (target) image and a distractor image. **B**. Spike density waveform averaged from 20 trials aligned on fixation target onset. All other behavioral and stimulus markers are centered at their average time of occurrence and grey boxes indicate the first standard deviation in event time.

Approximately 60% of neurons within the IC have been shown to respond to not only sound ^17^, but to some extent visual- and saccade-related activity ^24, 25^. Inputs from the lateral nucleus of the IC and the nucleus of the brachium of the IC to the SC also exist ^26^. This pathway may be responsible in part for the auditory activity observed in the deep layers of the SC ^27^ and is one route via which auditory information can influence saccadic eye movements. The response of IC neurons to visual stimulus and during eye movements is much less robust than the activity observed following visual stimulation and during saccades in the SC. The use of natural stimuli is expected to boost the responses of IC neurons in an audiovisual recall task to reveal multi-sensory integration that can influence saccade-related activity. Figure 6 illustrates a neuron’s activity that is putatively considered reward-dependent ^28, 29^. The neuron was located rostral to the IC and deep to the region known to contain neurons controlling saccade-related activity in the SC. The neural activity was clearly phase-locked to the task, but was less obviously linked to auditory stimuli ( Figure 6A), contrary to what one would ordinarily expect in IC neurons (compare with Figure 4). This activity was not strictly linked to visual stimuli, nor was it saccade-related in terms of SC activity. The activity of this neuron seemed to indicate an expectation of reward that builds up based on successfully meeting task-related milestones ( Figure 6B). During the task and especially following the onset of the sound, very distinct differences existed in the firing pattern of this neuron compared to between the two conditions.

**Figure 7.**
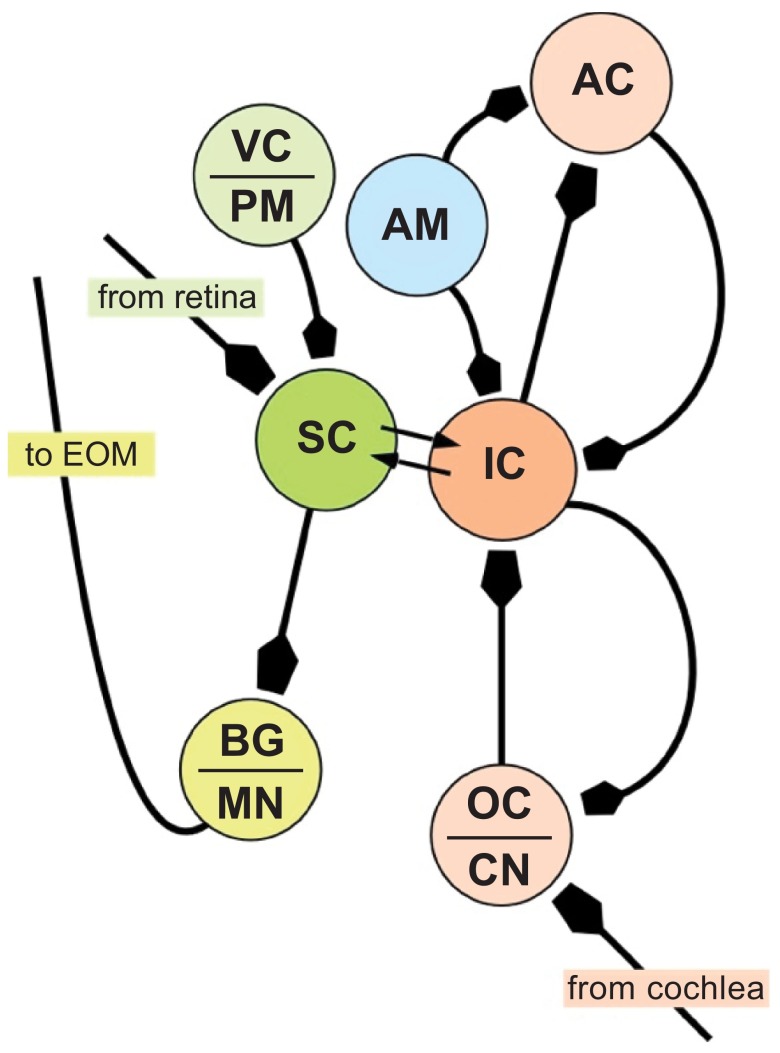
Schematic representing neural circuits creating visual-auditory interactions in the tectum. The superior (SC) and inferior colliculi (IC) receive direct visual (shades of green) and auditory (shades of red) projections, respectively and have reciprocal connections with each other. The IC also receives emotive inputs from the amygdala (AM) either directly ^43^ or via reward circuitry in the ventral striatum ^44^, and has reciprocal connections with the auditory cortex (AC) for cognitive processing ^48^. Saccadic eye movements are controlled by outputs from the SC via local burst generators (BG) driving motor neurons innervating extraocular muscles (EOM). The SC receives information from the visual cortex and premotor neurons in the frontal cortex, particularly the frontal eye fields. AC = Auditory Cortex; CN = cochlear nucleus (VIIIn); MN = motor neurons (nuclei of cranial nerves III, IV and VI); PM = Pre-motor Cortex; VC = Visual Cortex.

Both auditory and visual activity in space is read out in the SC in a manner that is appropriate for generating accurate saccades to sounds and images, respectively, although visually evoked saccades have high velocity, greater precision and shorter reaction times than auditory evoked saccades ^4, 11, 30^. These and many other findings clearly indicate an extensive auditory input to the SC. Briefly, visual information from the retina drives the development of and maintains a spatial representation of auditory space in the IC ^31– 33^. This has been demonstrated in owls ^33, 34^ and is believed to be true in mammals. We presume that retinal inputs pass through the optic tectum and the superficial layers of the superior colliculus (SCs) before converging on auditory areas in the IC ( Figure 7). A pathway from the retina to SCs to IC is known to exist in mammals ^35^. Over the long term, the convergence of visual and auditory signals reinforces an enduring spatial map in the IC. Recently, many neurons within the IC (the brachium of the IC. The external capsule of the IC, and the core of the IC), have been shown to respond to not only sound ^17^, but to some extent visual- and saccade-related activity and in some cases responses are modifiable by reward ^24, 25^.

In many species, including humans, who rely predominantly on vision for their survival, auditory cues may trigger eye movements either for interaction with the environment or for communication with conspecifics. Many researchers point to the SC and IC as components of a multi-modal sensory integration system, where visual and auditory signals within the brain merge into a co-dependent representation of the world ^3– 5, 36, 37^. Neurophysiological and anatomical data support the idea that this linkage occurs only two or three synapses beyond the retina and auditory nerve. Signals sent out of the IC and SC are also fed-back onto their independent systems helping to modulate behavior (see Figure 7).

In summary, collecting behavioral and neural data using our suite of scripts and hardware together with subsequent analysis yielded new insights, providing strong evidence for the advantage of using a novel and customized paradigm. Our scripted user-interface demonstrated that pairing auditory and visual stimuli caused modest changes in activity throughout the trial period in a receptive neuron located deep within the SC. This was in contrast to the response of the same neuron presented with the same stimuli when the animal listened to them passively. The SC appears to be the site where sensory signals encoded in different frames of reference converge, and are translated into a common coordinate system commanding movement execution ^11^ (e.g. retinotopic-centered commands to resolve motor error). Integration of auditory and visual information also appears to occur at this site. A major cortico-collicular auditory projection suggests that the cortex may direct this integration via the IC, particularly during the learning phase ^38, 39^. After that, subcortical circuits may function autonomously for computing a reaction.

## Conclusions

In conclusion, we have developed a simple and relatively straightforward user-interface that directs and monitors subject behavior as well as acquires data. This particular set-up and the customized paradigms used in this experiment may be impossible for vendors of commercial stimulus presentation and data acquisition software and hardware to develop for the general neuroscience community due to the specific needs of each research laboratory. Our experimental design and custom scripts, however, are flexible to meet virtually all experimental control and data acquisition needs of those interested in conducting behaviorally controlled, response-based experiments. We have used a modified design to run psychophysics experiments on human subjects and these can be combined with dense array EEG recordings in response to the presentation of auditory and visual stimuli ^40^. In essence, our template can be used to build any type of subject-interactive experiments. There is high potential for applying our pragmatic design to control neurobehavioral experiments using readily available hardware and software. Our studies, using earlier methodologies, showed that arousal has a role in bottom-up modulation of thalamic activity in the control of eye-movements ^41, 42^. Our new methodology allowed us to discover the location of audiovisual neurons at which reward-based, and possibly anxiety-driven, influences may converge to modulate behavior ^43– 47^. Studying these circuits in intact, normal animals is important to decipher the interplay of excitation and inhibition between different neural circuits for dynamic control of eye movement and gaze control.
