# Supplementary material for: A flexible user-interface for audiovisual presentation and interactive control in neurobehavioral experiments
Source: F1000Res. 2013 Jun 6;2:20. Originally published 2013 Jan 23. [Version 2] doi: 10.12688/f1000research.2-20.v2 (PMC3907162; doi:10.12688/f1000research.2-20.v2)
Supplement: Presentation script files. — The single necessary presentation script needed to display images and sounds upon command from the Spike2 control script. [file f1000research-2-1496-s0001.tgz › Presentation_Script.docx]

no_logfile = true;

default_font_size = 24;

# example of making presentation use visual angle in degrees

screen_width = 1280;

screen_height = 1024;

screen_bit_depth = 32;

# set these to screen measurements in inches

#screen_width_distance = 35.5;

screen_width_distance = 47.5;

screen_height_distance = 26.75;

# set screen_distance to distance between screen and eyes of subject

screen_distance = 48;

begin;

#picture {} default;

trial {

trial_duration = 1000;

picture {

text { caption = " "; } text1;

x = 0; y = 0;

} pic1;

} trial1;

trial {

trial_duration = 2000;

picture {

text { caption = " "; } text2;

x = 0; y = 0;

} pic2;

} blackScreen;

sound { wavefile { filename = "C:\\Documents and Settings\\USER\\Desktop\\Behavioral Control\\pics_sounds\\angryMonkey_crop.wav"; preload = true; } s1; } snd;

trial {

#trial_duration = 15000;

picture {

ellipse_graphic{

ellipse_width =0.25; ellipse_height = 0.25; color = 0, 255, 0;

}ellipse1;

x = 0; y = 0;

} circle_fig;

duration = next_picture;

} GreenDot;

trial {

#trial_duration = 15000;

sound snd;

picture { text { caption = " "; } text3; x = 0; y = 0;} pic3;

duration = 1000;

} SoundTrial;

picture {

default_code = "a"; # Always use this code for pic1...

default_port_code = 1; # Always use this port_code for pic1...

# unless other values are given

bitmap { filename = "C:\\Documents and Settings\\USER\\Desktop\\Behavioral Control\\pics_sounds\\angryMonkey_crop.jpg";} bitmapa;

x = 0; y = 0;

} SeqPic;

picture {

default_code = "a"; # Always use this code for pic1...

default_port_code = 1; # Always use this port_code for pic1...

# unless other values are given

bitmap { filename = "C:\\Documents and Settings\\USER\\Desktop\\Behavioral Control\\pics_sounds\\human_crop.jpg";} bitmapOutLier; x = -100; y = 0;

bitmap { filename = "C:\\Documents and Settings\\USER\\Desktop\\Behavioral Control\\pics_sounds\\angryMonkey_crop.jpg";} bitmap2P; x = +100; y = 0;

ellipse_graphic{ ellipse_width =0.25; ellipse_height = 0.25; color = 0, 255, 0; }ellipse2P; x = 0; y = 0;

} GreenDot2Pic;

picture {

default_code = "a"; # Always use this code for pic1...

default_port_code = 1; # Always use this port_code for pic1...

# unless other values are given

bitmap { filename = "C:\\Documents and Settings\\USER\\Desktop\\Behavioral Control\\pics_sounds\\human_crop.jpg";} bitmap2D7; x = +6; y = -6;

bitmap { filename = "C:\\Documents and Settings\\USER\\Desktop\\Behavioral Control\\pics_sounds\\angryMonkey_crop.jpg";} bitmap2D8; x = 0; y = -6;

ellipse_graphic ellipse2P; x = 0; y = 0;

bitmap { filename = "C:\\Documents and Settings\\USER\\Desktop\\Behavioral Control\\pics_sounds\\Black_Spider_Monkey_crop.jpg";} bitmap2D1; x = -6; y = -6;

bitmap { filename = "C:\\Documents and Settings\\USER\\Desktop\\Behavioral Control\\pics_sounds\\Elephant_crop.jpg";} bitmap2D2; x = -6; y = 6;

bitmap { filename = "C:\\Documents and Settings\\USER\\Desktop\\Behavioral Control\\pics_sounds\\Lizard_crop.jpg";} bitmap2D3; x = 0; y = 6;

bitmap { filename = "C:\\Documents and Settings\\USER\\Desktop\\Behavioral Control\\pics_sounds\\Peacock_crop.jpg";} bitmap2D4; x = +6; y = 6;

bitmap { filename = "C:\\Documents and Settings\\USER\\Desktop\\Behavioral Control\\pics_sounds\\Leopard_crop.jpg";} bitmap2D5; x = +6; y = -6;

bitmap { filename = "C:\\Documents and Settings\\USER\\Desktop\\Behavioral Control\\pics_sounds\\Turtle_crop.jpg";} bitmap2D6; x = 0; y = -6;

# bitmap { filename = "C:\\Documents and Settings\\USER\\Desktop\\Behavioral Control\\pics_sounds\\angryMonkey_crop.jpg";} bitmap2D7; x = +900; y = 0;

} GreenDot2MPic;

picture {

default_code = "a"; # Always use this code for pic1...

default_port_code = 1; # Always use this port_code for pic1...

# unless other values are given

bitmap bitmap2D7; x = +6; y = -6;

bitmap bitmap2D8; x = 0; y = -6;

bitmap bitmap2D1; x = +300; y = 0;

bitmap bitmap2D2; x = +400; y = 0;

bitmap bitmap2D3; x = +500; y = 0;

bitmap bitmap2D4; x = +600; y = 0;

bitmap bitmap2D5; x = +700; y = 0;

bitmap bitmap2D6; x = +800; y = 0;

#bitmap bitmap2D7; x = +900; y = 0;

} SeqMPic;

trial {

picture GreenDot2MPic;

duration = next_picture;

} GreenDotMPicTrial;

trial {

picture SeqMPic;

duration = next_picture;

} SeqMPicTrial;

picture {

default_code = "a"; # Always use this code for pic1...

default_port_code = 1; # Always use this port_code for pic1...

# unless other values are given

bitmap bitmapOutLier; x = -100; y = 0;

bitmap bitmap2P; x = +100; y = 0;

} Seq2Pic;

trial {

picture GreenDot2Pic;

duration = next_picture;

} GreenDot2PicTrial;

trial {

picture Seq2Pic;

duration = next_picture;

} Seq2PicTrial;

trial {

#trial_duration = 15000;

stimulus_event { sound snd; } events1;

picture circle_fig;

duration = next_picture;

} GreenDotSound;

trial {

# wavefile s1;

stimulus_event {

sound snd;

} events2;

picture SeqPic;

duration = next_picture;

} PicnSound;

trial {

#trial_duration = 15000;

picture {

ellipse_graphic{

ellipse_width =0.25; ellipse_height =0.25; color = 0, 255, 0;

}ellipse6;

x = 0; y = 0;

ellipse_graphic {

ellipse_width =0.25; ellipse_height =0.25; color = 0, 255, 0;

}ellipse2; x = -266; y = +100;

ellipse_graphic {

ellipse_width =0.25; ellipse_height =0.25; color = 0, 255, 0;

}ellipse3; x = -266; y = +80;

ellipse_graphic {

ellipse_width =0.25; ellipse_height = 0.25; color = 0, 255, 0;

}ellipse4; x = -266; y = -80;

ellipse_graphic {

ellipse_width =0.25; ellipse_height = 0.25; color = 0, 255, 0;

}ellipse5; x = -266; y = -100;

} circle_fig2;

duration = 1000;

} Green5Dot;

picture {

default_code = "a"; # Always use this code for pic1...

default_port_code = 1; # Always use this port_code for pic1...

# unless other values are given

ellipse_graphic{

ellipse_width =0.25; ellipse_height = 0.25; color = 0, 255, 0;

}ellipse7; x = 0; y = 0;

ellipse_graphic {

ellipse_width =0.25; ellipse_height = 0.25; color = 0, 255, 0;

}ellipse8; x = -266; y = +70;

ellipse_graphic {

ellipse_width =0.25; ellipse_height = 0.25; color = 0, 255, 0;

}ellipse9; x = -266; y = +80;

ellipse_graphic {

ellipse_width =0.25; ellipse_height = 0.25; color = 0, 255, 0;

}ellipse10; x = -266; y = -70;

ellipse_graphic {

ellipse_width =0.25; ellipse_height = 0.25; color = 0, 255, 0;

}ellipse11; x = -266; y = -100;

bitmap { filename = "C:\\Documents and Settings\\USER\\Desktop\\Behavioral Control\\pics_sounds\\angryMonkey_crop.jpg";} bitmapd;

x = -266; y = 0;

} SeqPic2;

begin_pcl;

int selN = 3;

int comError = 0;

array <int> selIDs[3] = { 3, 4 , 8 };

array <string> targetIDs[1];

array <string> soundName[1];

array <int> soundID[1];

array <int> randID[1];

array <string> picsName[1];

array <int> picsID[1];

int curI = 1; int history = 0;

array <double> CoorX[8] = { -7.5, 0.0, 7.5, -7.5, 7.5, -7.5, 0.0, 7.5 };

array <double> CoorY[8] = { 5.0, 5.0, 5.0, 0.0, 0.0, -5.0, -5.0, -5.0 };

/*Display message on black screen*/

sub

show_text( string message )

begin

text1.set_caption( message );

text1.redraw();

trial1.present()

end;

/*read name of stimuli to present from file on disc*/

sub

read_Files( string filePath,array <string,1> & tempdata2,array <int,1> & tempIDdata2)

begin

input_file in = new input_file;

in.open(filePath); in.set_delimiter( '\n' ); # for get_line()

string d_size=in.get_string();

int dataSize = int(d_size);

string junkVal=in.get_line();

loop int count = 1; until in.end_of_file() || count > dataSize || !in.last_succeeded()

begin

tempdata2.resize(count); tempIDdata2.resize(count);

tempdata2[count] = in.get_line();

tempIDdata2[count] = count; # setting picture ID

#term.print(tempdata2[count] + string(count)+"of total : "+d_size+"\n");

count = count + 1;

end;

end;

/*read \n terminated string$*/

sub

string read_anyword(input_port portin)

begin

#listen to arrival of data

string codes_string=""; int code_byte=0; int i=0; int limIndex=portin.total_count();

#term.print("visiting read_anyword");

#show_text("visiting read_anyword"+string(limIndex));

loop until code_byte==10 || i>=limIndex begin

i = i+1;

codes_string.resize(i);

code_byte = portin.codes(i);

codes_string.set_char(i,code_byte);

end;

if code_byte == 10 then

portin.clear(1,i);

return codes_string;

end;

#show_text("received string:"+codes_string+"& length is"+string(codes_string.count()));

return "";

end;

/*read data*/

sub

int read_word(input_port portin, string awaitS)

begin

string codes_string="";

loop until codes_string == awaitS

begin

codes_string=read_anyword(portin)

end;

portin.clear();

return 1;

end;

sub

int PresentStimulus(string fname,string sName)

begin

bitmapa.unload();

bitmapa.set_filename(fname);

#bitmapa.set_load_size(0.0,set_load_size(0.0,0.0,0.75);

bitmapa.load();

s1.unload();

s1.set_filename(sName);

s1.load();

#term.print("Trial Start ");

#term.print(sName+":");

#term.print(fname+"\n");

PicnSound.present();

return 1;

end;

sub

int PresentStimulusLR(string fname,string sName)

begin

bitmapa.unload();

bitmapa.set_filename(fname);

#bitmapa.set_load_size(0.0,0.0,0.75);

bitmapa.load();

#s1.unload();

#s1.set_filename(sName);

#s1.load();

#term.print("Trial Start ");

#term.print(sName+":");

#term.print(fname+"\n");

SeqPic.present();

return 1;

end;

sub

double readPosReal(input_port portin, string typeSt)

begin

string respText = "";

loop until respText.count()>0 begin

#show_text("wait X");

respText = read_anyword(portin);

#term.print("resp text is"+respText+"\n");

end;

respText.resize(respText.count()-1);

double xval = double(respText);

return xval;

end;

sub

int readPos(input_port portin, string typeSt)

begin

double xval = readPosReal(portin,typeSt);

double yval = readPosReal(portin,typeSt);

#show_text("Calibrating x @"+string(xval)+"and y @"+string(yval));

circle_fig.set_part_x(1,xval);

circle_fig.set_part_y(1,yval);

SeqPic.set_part_x(1,xval);

SeqPic.set_part_y(1,yval);

return 1;

end;

sub

int PresentStimulus2PG(input_port portin, output_port port, string fname, string tokenVal)

begin

double bitmap2P_X=readPosReal(portin,tokenVal);

double bitmap2P_Y=readPosReal(portin,tokenVal);

GreenDot2Pic.set_part_x(1,-bitmap2P_X);

GreenDot2Pic.set_part_y(1,bitmap2P_Y);

GreenDot2Pic.set_part_x(3,0); # GREEN DOT IN CENTER

GreenDot2Pic.set_part_y(3,0);

GreenDot2Pic.set_part_x(2,bitmap2P_X); # CORRECT IMAGE

GreenDot2Pic.set_part_y(2,bitmap2P_Y);

port.send_string(tokenVal+"_ACK\n");

GreenDot2PicTrial.present();

return 1;

end;

sub ############### change it to set position of pics

int PresentStimulusMPG(input_port portin, output_port port, string fname, string tokenVal)

begin

double bitmap2P_X=readPosReal(portin,tokenVal);

double bitmap2P_Y=readPosReal(portin,tokenVal);

#OUTLIER FIRST

GreenDot2MPic.set_part_x(1,CoorX[soundID[1]]);

GreenDot2MPic.set_part_y(1,CoorY[soundID[1]]);

#GreenDot2MPic.set_part_x(1,-bitmap2P_X);

GreenDot2MPic.set_part_x(3,0); # GREEN DOT IN CENTER

GreenDot2MPic.set_part_y(3,0);

GreenDot2MPic.set_part_x(2,CoorX[soundID[2]]); # CORRECT IMAGE

GreenDot2MPic.set_part_y(2,CoorY[soundID[2]]);

#distractors setting

GreenDot2MPic.set_part_x(4,CoorX[soundID[3]]);

#GreenDot2MPic.set_part_x(5,0);

GreenDot2MPic.set_part_x(5,CoorX[soundID[4]]);

GreenDot2MPic.set_part_x(6,CoorX[soundID[5]]);

#GreenDot2MPic.set_part_x(8,0);

GreenDot2MPic.set_part_x(7,CoorX[soundID[6]]);

GreenDot2MPic.set_part_x(8,CoorX[soundID[7]]);

GreenDot2MPic.set_part_x(9,CoorX[soundID[8]]);

GreenDot2MPic.set_part_y(4,CoorY[soundID[3]]);

GreenDot2MPic.set_part_y(5,CoorY[soundID[4]]);

GreenDot2MPic.set_part_y(6,CoorY[soundID[5]]);

GreenDot2MPic.set_part_y(7,CoorY[soundID[6]]);

GreenDot2MPic.set_part_y(8,CoorY[soundID[7]]);

GreenDot2MPic.set_part_y(9,CoorY[soundID[8]]);

#GreenDot2MPic.set_part_y(8,-distractor_X);

#GreenDot2MPic.set_part_y(9,-distractor_X);

GreenDot2MPic.present();

port.send_string(tokenVal+"_ACK\n"+string(CoorX[selIDs[curI]])+"\n"+string(CoorY[selIDs[curI]])+"\n");

return 1;

end;

sub ############### change it to set position of pics

int PresentStimulusMP(input_port portin,output_port port,string tokenVal)

begin

double bitmap2P_X=readPosReal(portin,tokenVal);

double bitmap2P_Y=readPosReal(portin,tokenVal);

SeqMPic.set_part_x(1,CoorX[soundID[1]]);

SeqMPic.set_part_y(1,CoorY[soundID[1]]);

SeqMPic.set_part_x(2,CoorX[soundID[2]]);

SeqMPic.set_part_y(2,CoorY[soundID[2]]);

SeqMPic.set_part_x(3,CoorX[soundID[3]]);

#SeqMPic.set_part_x(5,0);

SeqMPic.set_part_x(4,CoorX[soundID[4]]);

SeqMPic.set_part_x(5,CoorX[soundID[5]]);

#SeqMPic.set_part_x(8,0);

SeqMPic.set_part_x(6,CoorX[soundID[6]]);

SeqMPic.set_part_x(7,CoorX[soundID[7]]);

SeqMPic.set_part_x(8,CoorX[soundID[8]]);

SeqMPic.set_part_y(3,CoorY[soundID[3]]);

SeqMPic.set_part_y(4,CoorY[soundID[4]]);

SeqMPic.set_part_y(5,CoorY[soundID[5]]);

SeqMPic.set_part_y(6,CoorY[soundID[6]]);

#SeqMPic.set_part_y(8,-distractor_Y);

#SeqMPic.set_part_y(9,-distractor_Y);

SeqMPic.set_part_y(7,CoorY[soundID[7]]);

SeqMPic.set_part_y(8,CoorY[soundID[8]]);

port.send_string(tokenVal+"_ACK\n");

SeqMPicTrial.present();

return 1;

end;

sub

int PresentStimulus2P(input_port portin,output_port port, string fname,string tokenVal)

begin

double bitmap2P_X=readPosReal(portin,tokenVal);

double bitmap2P_Y=readPosReal(portin,tokenVal);

#setting image positions

Seq2Pic.set_part_x(1,-bitmap2P_X);

Seq2Pic.set_part_y(1,bitmap2P_Y);

Seq2Pic.set_part_x(2,bitmap2P_X);

Seq2Pic.set_part_y(2,bitmap2P_Y);

port.send_string(tokenVal+"_ACK\n");

Seq2PicTrial.present();

return 1;

end;

sub int initialization

begin

# read corresponding wave files

# read corresponding pic files

read_Files("C:\\Documents and Settings\\USER\\Desktop\\Behavioral Control\\object_lists\\picture_list.txt",picsName,picsID);

read_Files("C:\\Documents and Settings\\USER\\Desktop\\Behavioral Control\\object_lists\\sound_list.txt",soundName,soundID);

# if mode is shuffled presentation, Shuffle order & output new order of pics

randID.resize(picsID.count());

loop int ik=1; until ik > picsName.count() begin

term.print(string(ik)+"th is No."+string(picsID[ik])+": "+picsName[soundID[ik]]+"\n");

randID[ik]=ik;

ik=ik+1;

end;

#picsID.shuffle();

soundID = picsID;

randID = soundID;

# initialize multipic placement according to the text file on desk

bitmap2D7.unload();

bitmap2D7.set_filename(picsName[soundID[1]]);

bitmap2D7.load();

bitmap2D8.unload();

bitmap2D8.set_filename(picsName[soundID[2]]);

bitmap2D8.load();

bitmap2D1.unload();

bitmap2D1.set_filename(picsName[soundID[3]]);

bitmap2D1.load();

bitmap2D2.unload();

bitmap2D2.set_filename(picsName[soundID[4]]);

bitmap2D2.load();

bitmap2D3.unload();

bitmap2D3.set_filename(picsName[soundID[5]]);

bitmap2D3.load();

bitmap2D4.unload();

bitmap2D4.set_filename(picsName[soundID[6]]);

bitmap2D4.load();

bitmap2D5.unload();

bitmap2D5.set_filename(picsName[soundID[7]]);

bitmap2D5.load();

bitmap2D6.unload();

bitmap2D6.set_filename(picsName[soundID[8]]);

bitmap2D6.load();

# if mode is N-shuffle presentation, Shuffle order N pics whose ID has been sent & output new order of pics

return 1;

end;

sub int randDist(int targetIDIndex)

begin

int distractorIDIndex=targetIDIndex;

# if mode is shuffled presentation, Shuffle order & output new order of pics

randID.shuffle();

loop int i=1 until soundID[distractorIDIndex] != selIDs[targetIDIndex] begin

distractorIDIndex=randID[i];

i=1+1;

end;

term.print("Distractor ID "+string(soundID[distractorIDIndex])+"\n"+"Target ID "+string(soundID[targetIDIndex])+"\n");

return distractorIDIndex;

end;

sub int randDistLoad(int targetIDIndex)

begin

int distractorIDIndex = randDist(targetIDIndex);

bitmapOutLier.unload();

#bitmap2P.set_load_size(0.0,0.0,0.75);

bitmapOutLier.set_filename(picsName[distractorIDIndex]);

bitmapOutLier.load();

return 1;

end;

# read Nread\n,shuffle\nORNoshuffle, selN, selIDs for assocPic, LRPic, 2PIC, MPIC [MPIC not required but reading only for comaptibility]

sub int readStimuli(input_port portin,output_port port)

begin

string sceneType = read_anyword(portin);

term.print("kahana\n");

comError = 0;

loop until read_word(portin, "scenetype\n") == 1 begin

term.print("waiting scenetype");

comError = comError+1;

if (comError == 50000) then

port.send_string("commError_scenetype\n");

end;

end;

selN = int(readPosReal(portin,""));

selIDs.resize(selN);

term.print("Total "+string(selN)+"\n");

port.send_string("scenetype_ACK\n");

comError = 0;

loop int i=1 until i>selN begin

#reading single ID

int paRhliya=0;

loop until paRhliya>0 begin

paRhliya = int(readPosReal(portin,""));

#term.print(string(i)+"\n"+"length SelIDs"+string(selIDs.count()));

selIDs[i] = paRhliya;

term.print("waiting StimulusID\n");

comError = comError+1;

if (comError == 50000) then

port.send_string("commError_StimulusID\n");

end;

end;

term.print("Stimuli "+string(i)+" is: "+string(selIDs[i])+"\n");

term.print("Counting data\n");

port.send_string("numb_ACK\n");

i=i+1;

end;

#term.print("\nkaahaan ho");

return 1;

end;

if (output_port_manager.port_count() == 0 && input_port_manager.port_count() == 0 ) then

show_text( "No output ports selected!" );

else

#Port initialization

output_port port = output_port_manager.get_port( 1 );

input_port portin = input_port_manager.get_port( 1 );

#port.set_pulse_width( 1 );

output_port portLTP = output_port_manager.get_port( 2 );

portLTP.set_pulse_width( 1 );

#Seq2Pic.present();

#GreenDot2Pic.present();

#List of globals

/*Random presentation with picture ID sending*/

#port.send_string(string(picsID[1])+"\n");

/* Present black screen, then green dot */

#GreenDot.present();

#PresentStimulus(picsName[picsID[1]]);

#Present pictures one-by-one

int i=0;

loop until 1==0 begin

#show_text("Loop No: "+string(i));

string responesText = read_anyword(portin);

#intialization phase

if responesText=="startr\n" then

#show_text("strating");

picsID.resize(1); picsName.resize(1); i=1;

circle_fig.set_part_x(1,0);

circle_fig.set_part_y(1,0);

if initialization() == 1 then

port.send_string("start_ACK\n");

portLTP.send_code( 255 );

readStimuli(portin,port);

# read Nread\n,shuffle\nORNoshuffle, selN, selIDs for assocPic, LRPic, 2PIC, MPIC [MPIC not required but reading only for comaptibility]

# shuffle or no shuffle... sets shuffleFlag=1, shuffles selIDs array

# send ACK "Nread_ACK"

blackScreen.present();

#GreenDot.present();

# add utility to send "time of presentation"

#show_text("Received list of pictures and sent ACK back");

else

show_text("Error in Initialization");

port.send_string("Start_ERR\n"); # add utility to send "time of presentation"

end;

# Present next image

elseif responesText=="next\n" then

#show_text(responesText);

if i<selIDs.count() then

i=i+1;

else

i=1;

selIDs.shuffle();

# shuffle seldIDs array if shuffleFlag=1

end;

#show_text("Present Image"); #

readPos(portin,"next");

#port.send_string(string(picsID[i])+"\n");

port.send_string("next_ACK\n");

portLTP.send_code( 255 );

PresentStimulus(picsName[selIDs[i]],soundName[selIDs[i]]);

#term.print(picsName[picsID[i]]+"\n");

#portin.clear();

# Present same image

elseif responesText=="back\n" then

#break;

#show_text("Re-start the loop... as Spike2 asks to come back \n bcaz monkey ignores the dot");

#show_text(responesText);

readPos(portin,"back");

#port.send_string(string(picsID[i])+"\n");

port.send_string("back_ACK\n");

portLTP.send_code( 255 );

PresentStimulus(picsName[selIDs[i]],soundName[selIDs[i]]);

#portin.clear();

# Present green Dot

elseif responesText=="gren\n" then

#show_text(responesText);

readPos(portin,"gren");

port.send_string("gren_ACK\n");

portLTP.send_code( 255 );

GreenDot.present();

#port.send_string(string(100)+"\n");

#show_text("Pictures Finished!!!! at i value:"+string(i));

#port.send_string("end_ACK\n");

# Stop presenting

elseif responesText=="stop\n" then

port.send_string("stop_ACK\n");

portLTP.send_code( 255 );

show_text("Asked to be stopped");

show_text(" ");

# Erroneous token presenting

elseif responesText=="blak\n" then

port.send_string("blak_ACK\n");

portLTP.send_code( 255 );

blackScreen.present();

#show_text("Nothing received");

#present old stuff again

# Erroneous token presenting

elseif responesText=="cali\n" then

readPos(portin,"gren");

#read coordinates for green dot

port.send_string("cali_ACK\n");

portLTP.send_code( 255 );

GreenDot.present();

#show_text("Calibrating");

elseif responesText=="nextLR\n" then

readPos(portin,"nextLR");

port.send_string("nextLR_ACK\n");

portLTP.send_code( 255 );

PresentStimulusLR(picsName[selIDs[i]],soundName[selIDs[curI]]);

elseif responesText=="backLR\n" then

readPos(portin,"backLR");

port.send_string("backLR_ACK\n");

portLTP.send_code( 255 );

PresentStimulusLR(picsName[selIDs[i]],soundName[selIDs[curI]]);

elseif responesText=="GNSDn\n" then

if i<selIDs.count() then

i=i+1;

else

i=1;

selIDs.shuffle();

end;

curI = i;

#show_text(responesText);

s1.unload();

s1.set_filename(soundName[selIDs[curI]]);

s1.load();

#code changed for MPIC

readPos(portin,"GNSDn");

port.send_string("GNSDn_ACK\n");

portLTP.send_code( 255 );

GreenDotSound.present();

#Change target picture for anticipated change

bitmap2P.unload();

bitmap2P.set_filename(picsName[selIDs[curI]]);

bitmap2P.load();

elseif responesText=="GNSDb\n" then

#show_text(responesText);

readPos(portin,"GNSDb");

s1.unload();

s1.set_filename(soundName[selIDs[i]]);

s1.load();

#code changed for MPIC

curI = i;

port.send_string("GNSDb_ACK\n");

portLTP.send_code( 255 );

GreenDotSound.present();

#Change target picture for anticipated change

bitmap2P.unload();

bitmap2P.set_filename(picsName[selIDs[curI]]);

bitmap2P.load();

elseif responesText=="G2PC\n" then

if history != curI then

randDistLoad(curI);

history =curI;

end;

PresentStimulus2PG(portin,port,picsName[selIDs[curI]],"G2PC");

portLTP.send_code( 255 );

elseif responesText=="next2P\n" then

PresentStimulus2P(portin,port,picsName[selIDs[curI]],"next2P");

portLTP.send_code( 255 );

elseif responesText=="back2P\n" then

PresentStimulus2P(portin,port,picsName[selIDs[curI]],"back2P");

portLTP.send_code( 255 );

elseif responesText=="GMPC\n" then

PresentStimulusMPG(portin,port,picsName[selIDs[curI]],"GMPC");

portLTP.send_code( 255 );

elseif responesText=="nextMP\n" then

curI = i;

PresentStimulusMP(portin,port,"nextMP");

portLTP.send_code( 255 );

elseif responesText=="backMP\n" then

curI = i;

PresentStimulusMP(portin,port,"backMP");

portLTP.send_code( 255 );

elseif responesText=="testing\n" then

double xt=readPosReal(portin,"testing");

double yt=readPosReal(portin,"testing");

double Tt=readPosReal(portin,"testing");

double Bt=readPosReal(portin,"testing");

double Lt=readPosReal(portin,"testing");

double Rt=readPosReal(portin,"testing");

SeqPic2.set_part_x(6,xt);

SeqPic2.set_part_y(6,yt);

show_text("Pic x:"+string(xt)+"Pic y:"+string(yt));

SeqPic2.set_part_x(2,xt);

SeqPic2.set_part_y(2,Tt);

show_text("Pic topx:"+string(xt)+"Pic topy:"+string(Tt));

SeqPic2.set_part_x(3,xt);

SeqPic2.set_part_y(3,Bt);

show_text("Pic Botx:"+string(xt)+"Pic Boty:"+string(Bt));

SeqPic2.set_part_x(4,Lt);

SeqPic2.set_part_y(4,yt);

show_text("Pic Leftx:"+string(Lt)+"Pic Lefty:"+string(yt));

SeqPic2.set_part_x(5,Rt);

SeqPic2.set_part_y(5,yt);

show_text("Pic Rightx:"+string(Rt)+"Pic Righty:"+string(yt));

SeqPic2.set_part_x(1,xt);

SeqPic2.set_part_y(1,yt);

show_text("Pic Centerx:"+string(xt)+"Pic Centery:"+string(yt));

portLTP.send_code( 255 );

SeqPic2.present();

elseif responesText=="SNDDot\n" then

#show_text(responesText);

readPos(portin,"SNDDot");

port.send_string("SNDDot_ACK\n");

portLTP.send_code( 255 );

SoundTrial.present();

GreenDot.present();

#port.send_string(string(100)+"\n");

#show_text("Pictures Finished!!!! at i value:"+string(i));

#port.send_string("end_ACK\n");

# Stop presenting

else #<<<<<<<<<<<<<<<<<<<<<<<<<<<<<<<<<<<<<<<<<<<<<<<<<<<<<<<

term.print("Error Token : " + responesText + "\n"); #<<<<<<<<<<<<<<<<<<<<<<<<<<<<<<<<<

#blackScreen.present();

#show_text("Un-expected token received: "+responesText);

#show_text(" "+responesText);

#show_text(" ");

#return 0;

end; #end of if

end;#end of loop

end;#end of main if
