# Supplementary figures and images for: A flexible user-interface for audiovisual presentation and interactive control in neurobehavioral experiments
Source: F1000Res. 2013 Jun 6;2:20. Originally published 2013 Jan 23. [Version 2] doi: 10.12688/f1000research.2-20.v2 (PMC3907162; doi:10.12688/f1000research.2-20.v2)

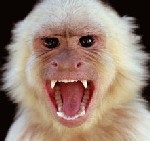

Supplement: Associated sound and target image files. — Paired sound and image files necessary for running all association tasks, frequency tone ranges used for testing frequency tuning of neurons (not discussed in the text) and species-specific communication calls. [file f1000research-2-1496-s0000.tgz › angryMonkey_crop.jpg]

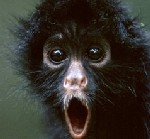

Supplement: Associated sound and target image files. — Paired sound and image files necessary for running all association tasks, frequency tone ranges used for testing frequency tuning of neurons (not discussed in the text) and species-specific communication calls. [file f1000research-2-1496-s0000.tgz › Black_Spider_Monkey_crop.jpg]

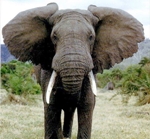

Supplement: Associated sound and target image files. — Paired sound and image files necessary for running all association tasks, frequency tone ranges used for testing frequency tuning of neurons (not discussed in the text) and species-specific communication calls. [file f1000research-2-1496-s0000.tgz › Elephant_crop.jpg]

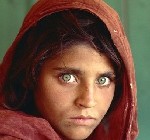

Supplement: Associated sound and target image files. — Paired sound and image files necessary for running all association tasks, frequency tone ranges used for testing frequency tuning of neurons (not discussed in the text) and species-specific communication calls. [file f1000research-2-1496-s0000.tgz › human_crop.jpg]

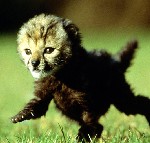

Supplement: Associated sound and target image files. — Paired sound and image files necessary for running all association tasks, frequency tone ranges used for testing frequency tuning of neurons (not discussed in the text) and species-specific communication calls. [file f1000research-2-1496-s0000.tgz › Leopard_crop.jpg]

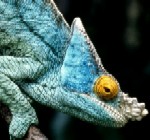

Supplement: Associated sound and target image files. — Paired sound and image files necessary for running all association tasks, frequency tone ranges used for testing frequency tuning of neurons (not discussed in the text) and species-specific communication calls. [file f1000research-2-1496-s0000.tgz › Lizard_crop.jpg]

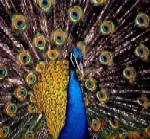

Supplement: Associated sound and target image files. — Paired sound and image files necessary for running all association tasks, frequency tone ranges used for testing frequency tuning of neurons (not discussed in the text) and species-specific communication calls. [file f1000research-2-1496-s0000.tgz › Peacock_crop.jpg]

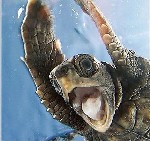

Supplement: Associated sound and target image files. — Paired sound and image files necessary for running all association tasks, frequency tone ranges used for testing frequency tuning of neurons (not discussed in the text) and species-specific communication calls. [file f1000research-2-1496-s0000.tgz › Turtle_crop.jpg]
