# Supplementary material for: A flexible user-interface for audiovisual presentation and interactive control in neurobehavioral experiments
Source: F1000Res. 2013 Jun 6;2:20. Originally published 2013 Jan 23. [Version 2] doi: 10.12688/f1000research.2-20.v2 (PMC3907162; doi:10.12688/f1000research.2-20.v2)
Supplement: Peer review history

# Peer review: Referee response for version 2

**Published:** 2013-08-29

**Authors:** Bruce Cumming

**Affiliations:** 1 Laboratory of Sensorimotor Research, National Eye Institute, National Institutes of Health (NIH), Bethesda, MD, USA

This would be a much stronger contribution if the authors added measurements of the critical parameters, including quality of video synchronization and loop delay. Their responses place some theoretical limits, but a measurement would be much better.

Nonetheless, as a forum for publishing code that can be used by people not concerned with these details, this may be useful.

I have read this submission. I believe that I have an appropriate level of expertise to confirm that it is of an acceptable scientific standard.

# Peer review: Referee response for version 2

**Published:** 2013-07-09

**Authors:** Farrel Robinson

**Affiliations:** 1 Department of Biological Structure, University of Washington, Seattle, WA, USA

The amendments made to the article have improved the manuscript.

I have read this submission. I believe that I have an appropriate level of expertise to confirm that it is of an acceptable scientific standard.

# Peer review: Referee response for version 1

**Published:** 2013-05-23

**Authors:** Farrel Robinson

**Affiliations:** 1 Department of Biological Structure, University of Washington, Seattle, WA, USA

This title is appropriate. The content of this article clearly describes the authors’ interface, components, and connections and the behaviour of this system. The authors support their assertion that they have developed a flexible and useful system for controlling and recording behaviour experiments. The authors show enough appropriate examples to support their assertions.

I have read this submission. I believe that I have an appropriate level of expertise to confirm that it is of an acceptable scientific standard.

# Peer review: Referee response for version 1

**Published:** 2013-03-25

**Authors:** Vincent Ferrera

**Affiliations:** 1 Columbia Neuroscience, Columbia University Medical Center, New York, NY, USA

1. This paper describes an interesting but fairly standard set-up for neurophysiology data acquisition in behaving subjects. It would be nice to have more details about how timing and synchronization were verified, and also what are the special capabilities of the hardware that justifies their selection? Nevertheless, it is good to know what other people are using and this is therefore a useful contribution.
**Minor comments**
   
2. The Power 1401 is rather expensive. What special capabilities justify this cost?
   
3. Introduction: “which nevertheless continues to be the most reliable and useful way to understand neural computations and function.” This statement is debatable.
   
4. The communication between the spike2 and presentation computers appears critical. More details would be welcome. What exactly is conveyed by the 1-bit com port? What is the signal timing diagram? How is this bit manipulated?
   
5. A critical issue regarding the use of LCD displays for neurophysiology experiments is stimulus timing. How did the experimenters determine the relationship between the video output of the presentation computer and the actual appearance of stimuli on the display? 
   
6. Not clear about the hardware for neural signals. Was neural data stored on a third computer? Or on the spike2 computer? How many channels, what sampling rate, etc? How were spikes detected – window discriminator, waveform analysis?

I have read this submission. I believe that I have an appropriate level of expertise to confirm that it is of an acceptable scientific standard.

# Response

**Published:** 2013-08-22

**Authors:** Jagmeet Kanwal

**Affiliations:** Georgetown University, USA

Thank you for taking the time to review our manuscript. Below we provide our detailed response to specific comments per changes made in the updated version of the manuscript. 

1. We generally agree with the reviewer’s point. Our main objective was to document an example for the benefit of others, given that the F1000Research journal provides a mechanism for archiving stimuli, scripts and raw data that the research community at large can readily access. We provide additional details in the revised version of the manuscript as suggested by the reviewer. Specifically, we include information relating to our data collection sampling parameters, serial line communication timing, analog-to-digital converter (ADC) sampling rates and clock speeds for sequencer monitoring of behavior.
*Minor comments*
2. The technical specifications can be found at: http://www.ced.co.uk/pru.shtml. We now cite this resource in the revised version. We specifically choose the Power 1401 because the hardware is capable of converting analog signals at high (1MHz) rates while simultaneously reading the ADC channels to allow the user to monitor subject behavior. Thus, the behavioral monitoring script loaded into the 1401’s sequencer at a 1 µs tick rate; i.e., one line of code was read with each tick of the clock. Combining these two capabilities gave us practically instantaneous monitoring of our subject and the ability to respond to their behavior with sub-millisecond precision. In the examples presented, we simply rewarded the animal for maintaining its eye position within a defined spatiotemporal window. One could extend this monitoring by defining a threshold on eye velocity, using that threshold to output a TTL pulse to trigger an external stimulator with similar precision.
3. This statement now reads: *“which nevertheless continues to be* *one of* *the most reliable and useful ways to understand neural computations and function.*”
4. Bit-1 of the serial line, com port, conveys the hexadecimal representation of the words (descriptors and terminators) used to call images and sounds and response tokens between acquisition and presentation computers, respectively.  This bit was opened, written to, and closed by the respective portions of the scripts running on the acquisition or presentation computers. The signal passed between computers in less than a millisecond.  We are unclear on which “signal timing diagram” is referred to by the reviewer since we do not use this term in the manuscript.
5. We agree that LCD displays include an inherent error in the timing of the display of images because of their refresh rates (at 60Hz, the error could be up to ~16ms per frame). The error can be exacerbated by slow video rendering by the video card, which nowadays is less of an issue than in the past. We did not determine the exact error of our system. However, the response time of the display was 8 ms, which is half the refresh rate meaning that the system carries a maximum error of approximately 16 ms to refresh the entire image. For computing absolute perceptual or behavioral response delays, the refresh rate can be subtracted from the timing of the behavioral response, e.g., in our case eye-movements, though we were not interested in these particular parameters, only in using eye movements to control stimulus presentation and reward delivery.
6. The neural signal passed through a filter and amplifier before undergoing analog-to-digital conversion by the 1401. We stored the data on the same computer running the acquisition script. We typically collect one channel of raw neural signal at either 25kHz or 50kHz, four channels corresponding to horizontal and vertical eye position at 1kHz, one auditory channel at 25kHz, and one channel of timestamps at 10kHz, generated on-the-fly during acquisition of data using an adjustable threshold set on the channel collecting the neural data on spike timestamps. Digitizing the raw neural signal allowed for post-hoc analysis using the Spike2 software that provides software window discriminators and level detectors as well as various forms of waveform analysis including template matching and spike sorting using PCA algorithms.

# Peer review: Referee response for version 1

**Published:** 2013-03-21

**Authors:** Bruce Cumming

**Affiliations:** 1 Laboratory of Sensorimotor Research, National Eye Institute, National Institutes of Health (NIH), Bethesda, MD, USA

1. The authors describe their system for presenting audio-visual stimuli and making neural recordings, by combining two packages, 'Presentation' and 'Spike2'. It sounds like a very reasonable arrangement for their purposes. But they do not make a case that this system achieves anything special methodologically. People have been doing experiments similar to those they describe for many (20+) years with a variety of different systems. It seems that the advance here is that this system is easy to use/set up. This is very likely to be true provided users do substantially similar experiments. But that’s true for almost any system. How easy would it be to do something else? Without some way of demonstrating in some objective way what the range of the system is, it is impossible to say if there is real methodological contribution here. 
**Smaller questions**
2. An important problem is how precisely video events are synchronized with the neurophysiology. The moment at which a display computer requests an image change and the moment at which the first pixels of the new image are actually displayed on their LCD are two different things, and may well not even have a fixed delay (depending on the details of both the display and the rendering). How is this achieved? What is the delay? How variable is it? These crucial parameters are not reported.
3. What is the total loop delay from detecting some event in the A/D stream (eye movement, Spike) and the change in some output (electrical stimulus, image refresh with a new image)? If detecting these events depends upon the Spike2 control script, then delays can be quite long. Implementing them in the Sequencer is much harder. This loop delay potentially places fundamental limits on the range of applications that might be possible (whether gazed contingent displays are possible, or performing cancellation tests with antidromic stimulation). 
4. Since the paper objective is to describe a software/hardware system, the details about surgery and training, and most of the results, seem irrelevant. This space would be better used describing measures of the system performance. 
5. In principle one might do something similar combining other separate systems e.g. any other commercial electrophysiology system and psychophysics toolbox for the display. What are the merits of the different possibilities? Without comparing the available options, this description is of limited use.

I have read this submission. I believe that I have an appropriate level of expertise to state that I do not consider it to be of an acceptable scientific standard, for reasons outlined above.

# Response

**Published:** 2013-08-22

**Authors:** Jagmeet Kanwal

**Affiliations:** Georgetown University, USA

We thank the referee for taking out the time to review our manuscript and have addressed the issues raised in the revision. Below we respond to each comment. 

1. We offer those so inclined to use these two packages a clear, working set of scripts as an example that can be used as a springboard in the development of their own experiments.  In this way, our paper makes a significant methodological contribution, saving others precious time to focus on their experimental design. We were motivated by the F1000Research journal’s ability to provide a mechanism for archiving stimuli, scripts and raw data that the research community at large can readily access.
***Smaller questions*:**
2. We agree that LCD displays include an inherent error in the timing of the display of images because of their refresh rates (at 60Hz the error could be up to ~16ms per frame). The error can be exacerbated by slow video rendering by the video card, which nowadays is less of an issue than in the past. The delay in our system was ~16ms, but the exact error (undetermined) is likely much less. We now include information on these issues in the revised version of the manuscript.
3. The analog-to-digital converter (ADC) cycles through the analog signals at 1MHz (providing a read rate of 1 µs for the behavioral monitoring script loaded to the 1401’s sequencer); one line of code is acted upon with each tick of the clock. The sequencer has direct access to the ADC signals. The monitoring of events in this way is dependent on the length of the sequencer script loop. In our example, we sampled the position of the eye every 9 µs while in the loop. This means reading even a couple of hundred lines of code takes an order of magnitude less time for loop delays, allowing implementation of cancellation tests with antidromic stimulation, if needed.  As indicated earlier, the delays of concern originate more with display issues for visual stimulation using naturalistic stimuli (simpler stimuli can be presented by other means), but these are still much smaller than perceptual delays, which are on the order of a couple of hundred milliseconds. For perturbing subconscious perception, either display delays could be accounted for in a stimulation paradigm or an LCD display may be substituted with a motorized slide projector. 
4. We describe relevant details regarding system performance as well as offer the results obtained in our study as a proof of concept, highlighting a potential scientific advance that may not be possible with a more cumbersome system. 
5. We agree that there are many options available for conducting neurophysiological studies and sometimes making the best choice can be difficult. A comparison between specific options would be helpful, but is somewhat arbitrary in the absence of knowledge of all available equipment and experimental goals. Therefore, we simply provide one example for a specific set of experiments and leave it to the reader to make the necessary comparisons given their objectives and available equipment.  For this reason, we also believe it is important to illustrate the usage of the proposed setup in generating new findings and describe them adequately as well as provide animal protocols, as indicated by the editors/publishers. To that end, this manuscript accomplishes a clear and specific methodological goal.
